# Activation in the Right Inferior Parietal Lobule Reflects the Representation of Musical Structure beyond Simple Pitch Discrimination

**DOI:** 10.1371/journal.pone.0155291

**Published:** 2016-05-19

**Authors:** Isabelle Royal, Dominique T. Vuvan, Benjamin Rich Zendel, Nicolas Robitaille, Marc Schönwiesner, Isabelle Peretz

**Affiliations:** 1 Département de psychologie, Université de Montréal, Québec, Canada; 2 International Laboratory for Brain, Music and Sound Research (BRAMS), Université de Montréal, Québec, Canada; 3 Center of Research on Brain, Language and Music (CRBLM), McGill University, Québec, Canada; 4 Faculty of Medicine, Division of Community Health and Humanities, Memorial University of Newfoundland; UNLV, UNITED STATES

## Abstract

Pitch discrimination tasks typically engage the superior temporal gyrus and the right inferior frontal gyrus. It is currently unclear whether these regions are equally involved in the processing of incongruous notes in melodies, which requires the representation of musical structure (tonality) in addition to pitch discrimination. To this aim, 14 participants completed two tasks while undergoing functional magnetic resonance imaging, one in which they had to identify a pitch change in a series of non-melodic repeating tones and a second in which they had to identify an incongruous note in a tonal melody. In both tasks, the deviants activated the right superior temporal gyrus. A contrast between deviants in the melodic task and deviants in the non-melodic task (melodic > non-melodic) revealed additional activity in the right inferior parietal lobule. Activation in the inferior parietal lobule likely represents processes related to the maintenance of tonal pitch structure in working memory during pitch discrimination.

## Introduction

The human auditory system is highly sensitive to pitch, and many studies have documented how the brain processes pitch change. The standard approach has been the oddball paradigm, characterized by the presentation of sequences of repetitive identical auditory stimuli that are infrequently interrupted by a deviant stimulus that differs in pitch.

Using this paradigm, electroencephalographic (EEG) and magnetoencephalographic (MEG) studies have found that the brain’s automatic response to deviant stimuli is indexed by a response called the mismatch negativity (MMN) [1]. Whereas the MMN can be evoked when attention is directed away from the auditory environment, later components such as the P3a and P3b reflect the orientation of attention towards the deviant sound, and are elicited by the detection and evaluation of the deviant target. The P3a is thought to originate from frontal attention-orienting mechanisms as well as from the processing of novelty, whereas the P3b requires the stimulus to be task-relevant, and is thought to originate from the temporo-parietal activity associated with attention, and could be related to subsequent memory processing [2].

Functional magnetic resonance imaging (fMRI) has been used to investigate the specific brain regions activated by pitch oddball tasks, and several regions have been shown to be activated by the presentation of deviant sounds. These areas include the bilateral superior temporal gyri (STG), the right inferior frontal gyrus (IFG) as well as the right inferior parietal lobule (IPL) [3–5]. Moreover, the right STG has been found to be more sensitive to pitch change than the left STG [6], consistent with the notion that the right auditory cortex has a finer pitch resolution than the left [7–9].

The ability to detect pitch change is also critical for music perception. In a musical context, detecting a sour note requires establishing a mental representation of the tonal structure, and then recognizing a deviation. Interestingly, violations of more complex auditory sequences based on the Western tonal system, such as melodies or chord progressions evoke an automatic response, known as an Early Right Anterior Negativity (ERAN; [10,11]). Moreover, like pitch oddballs, tonal violations elicit a late, attention dependent response. This response, called the P600, is thought to reflect the integration of current pitches into the ongoing tonal context [12,13].

Previous studies have shown that the predominant generator of the ERAN is the inferior frontal gyrus [14]. Furthermore, tonally incongruous chords evoke increased bilateral activity in the STG and IFG [10,11,15], similar to the regions implicated in pitch change detection tasks performed in non-musical contexts. Accordingly, there is ample evidence linking specific brain regions with the processing of pitch changes, and functional studies contrasting the ERAN and MMN [16,17].

It is currently unclear whether any brain areas preferentially process pitch violations in musical contexts relative to pitch differences heard in non-musical contexts. The purpose of this study was to compare pitch change detection in these two distinct contexts in order to determine which brain areas are activated preferentially to pitch violations in music, beyond simple pitch discrimination.

We anticipated that processing pitch violations in musical (melodic) and non-musical (non-melodic) contexts would increase the blood oxygen level-dependent (BOLD) signal bilaterally in the STG and IFG. We expected to observe increased activation in the right STG during the melodic task as compared to the non-melodic task, as this area has been implicated in music-specific processing [18–20].

## Materials and Methods

### Participants

14 healthy right-handed participants were recruited and provided their written informed consent to participate in this study (M = 23.3 years ± 3.6 years; 5 males). None of the participants reported having undergone formal musical training, suffered from amusia, or had a history of neurological conditions, and all participants fulfilled the inclusion criteria for the safe use of fMRI. The research protocol was approved by the *Comité Mixte d’éthique de la recherche du Regroupement Neuroimagerie Québec (CMER-RNQ)* of the *Functional Neuroimaging Unit (UNF)* affiliated with the *Centre de recherche de l’Institut Universitaire de Gériatrie de Montréal (CRIUGM)*.

### Stimuli and behavioral procedure

#### Melodic task

**Fig 1:**
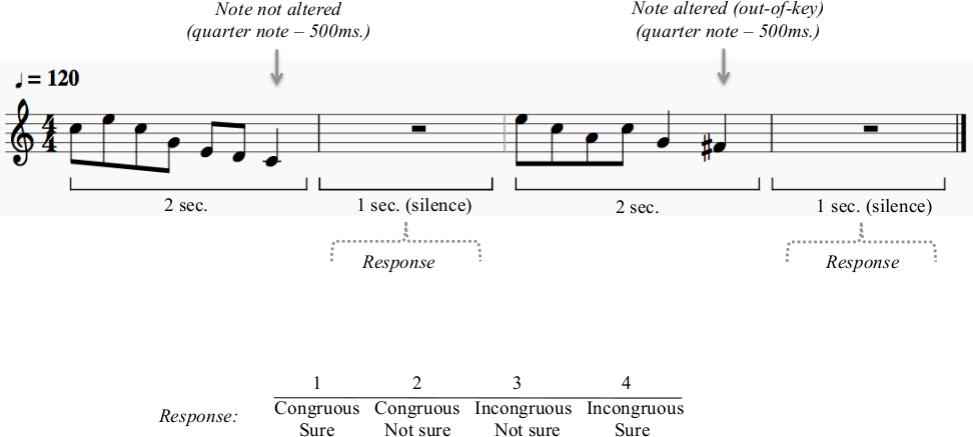
Melodic task. Half the melodies were in-tune, 25% were out-of-tune and 25% were out-of-key. The out-of-tune and out-of-key melodies were created by altering the last note of the in–tune melodies, by shifting them by either 50 cents (out-of-tune) or 100 cents (out-of-key). In the melodic task, participants judged whether a melody contained an incongruous note. Their judgment as well as their level of confidence were recorded for each trial on a four point scale (1-congruous, sure; 2-congruous, not sure; 3-incongruous, not sure; 4-incongruous, sure).

Stimuli for the melodic task consisted of 90 novel melodies, which were created using a synthesized piano timbre and a constant attack. Melodies were composed in the key of C and transposed to the keys of D and G, and ranged in pitch from F#3-B5 (F0 = 185–991 Hz). On average, each melody contained 8.6 tones and were presented at a tempo of 120 beats per minute. Each melody was one bar in length (four beats) and lasted for two seconds. There was one second of silence between the presentations of each melody (Fig 1). To create deviant stimuli, the original 90 melodies were modified so that the last tone (i.e., target tone) was altered in pitch. The modified tone was always the final note of the melody and was always 500 ms long (quarter note). *Out-of-tune* melodies were created by shifting the target tone by 50 cents, such that the note was not part of the chromatic nor diatonic scales. *Out-of-key* melodies were created by shifting the target tone by 100 cents (1 semitone), such that the note was part of the chromatic scale, but not part of the pitch set (diatonic scale) used for the melody. Accordingly, for the melodic task there were three stimulus types: *in-tune*, *out-of-key & out-of-tune* for a total of 810 possible stimuli (i.e. 90 melodies X 3 stimulus types X 3 keys).

In the melodic task, participants heard a melody and had to judge whether the last note was incongruous. Half of the presented melodies were *in-tune*, 25% were *out-of-tune* and 25% were *out-of-key*. The question was formulated in one sentence, presented on the screen. Their judgment as well as their level of confidence were recorded for each trial on a four point scale (1-congruous, sure; 2-congruous, not sure; 3-incongruous, not sure; 4-incongruous, sure) using two 2-button response boxes (one for each hand). Participants used the index and middle fingers on each hand to respond. Button-response mappings were counterbalanced across participants. Recoding of the responses was done in order to analyze accuracy and confidence separately. Participants’ reaction times were also measured. The melodic task consisted of 384 out of 810 possible melodies presented randomly.

#### Non-melodic task

**Fig 2:**
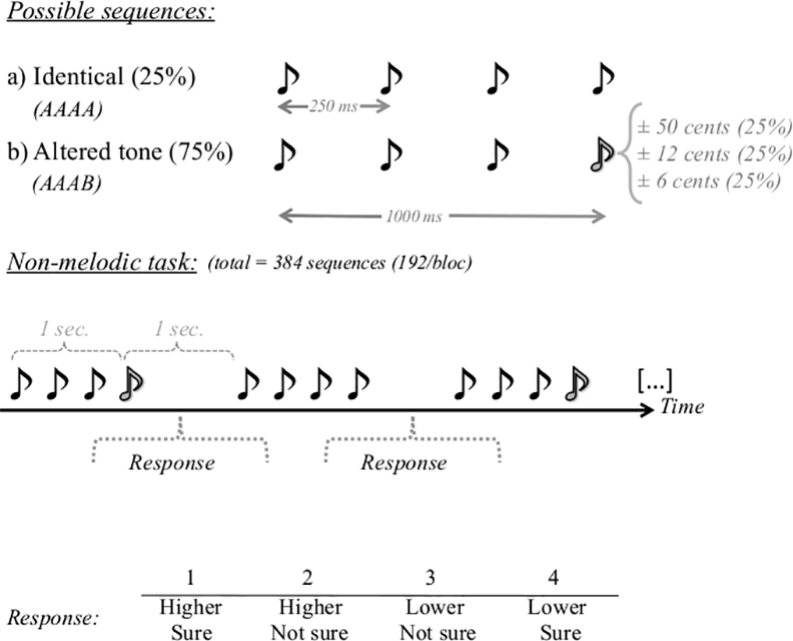
Non-melodic task. The non-melodic task consisted of four-tone sequences. The 4^th^ tone was either: identical to the preceding ones (25% of the trials); shifted by 6.25 cents (25% of the trials); shifted by 12.50 cents (25% of the trials); or shifted by 50 cents (25% of the trials). Participants judged whether the 4^th^ tone was higher or lower in pitch than the preceding tones. Judgment and confidence level were recorded on a four-point scale (1-higher, sure; 2-higher, not sure; 3-lower, not sure; 4-lower, sure).

Stimuli for the non-melodic task were sequences of four synthesized tones created by combining square and sine waves to create a piano timbre. Each tone had a duration of 250 ms (onset ramp = 30 ms, offset ramp = 20 ms) and the four tones were presented in succession without intervening silences, creating a sequence that lasted for one second (Fig 2). There was one second of silence between presentations of each sequence. The first three tones were always played at a pitch level of A4 (440 Hz), and the fourth tone (i.e., target tone) could either be identical (AAAA) or altered (AAAB) in pitch. The altered tones (B) were either higher or lower in pitch than the identical ones (A), with pitch differences of 6.25 cents (438 or 442 Hz), 12.5 cents (437 or 443 Hz) or 50 cents (427 or 453 Hz). Accordingly, for the non-melodic task there were four stimulus types: *identical*, *6*.*25 cents*, *12*.*5 cents & 50 cents*, and each stimulus type represented 25% of the trials.

In the non-melodic task, participants heard a sequence of four tones and had to judge whether the fourth tone was higher or lower in pitch than the preceding tones. The question was formulated in one sentence, presented on the screen. Their answers as well as their level of certainty were recorded on a four-point scale (1—higher, sure; 2—higher, not sure; 3—lower, not sure; 4—lower, sure) using two 2-button responses boxes (on in each hand). Button-response mappings were counterbalanced across participants. Recoding of the responses was done in order to analyze accuracy and confidence separately. Participants’ reaction times were also measured. The non-melodic task consisted of 384 sequences presented randomly.

### Imaging parameters

Anatomical T_1_-weighted images (3D MPRAGE: echo time = 3 ms, repetition time = 2300 ms, matrix size: 256 × 256 × 176, voxel size 1 × 1 × 1 mm) were acquired for every subject on a Siemens TIM Trio 3 Tesla MRI scanner using a 12-channel head-coil. Functional images were acquired using an echo-planar EPI T2* sequence (TR/TE = 2000/30 ms, flip angle = 75°, voxel size = 3 x 3 x 3.2 mm (28.8 mm^3^), FOV = 192 mm x 192 mm, 33 slices per volume).

### fMRI protocol

Tasks were administered with a computer running Matlab (The Mathworks Inc., Natick, MA, USA). Stimuli were presented using scanner-compatible earplugs with foam inserts, and the volume was set to a loudness that was comfortable for the participant before the scanning began. Melodic and non-melodic tasks were presented separately in sequence with the order of task presentation counterbalanced between participants (i.e. the order of blocks for each condition were alternated). In order to equalize the number of trials received of each task, participants were presented with 3 blocks of the melodic task (3 x 128 melodies = 384 trials) and 2 blocks of the non-melodic task (2 x 192 sequences = 384 trials). The different numbers of blocks for the two conditions were the consequence of the different trial lengths (i.e. 2s vs. 3s). 242 functional volumes were acquired in each block.

### Data processing and analyses

Functional data were analyzed using SPM8 (Statistical Parametric Mapping). For each individual, functional images were registered to the individual high-resolution anatomical image using linear registration and then registered to the ICBM152 standard brain for the group analysis using nonlinear registration. Functional data were motion-corrected using the motion correction in SPM8, high-pass filtered, preserving information below 128 Hz and spatially smoothed using a Gaussian kernel of 5 mm full width at half maximum. Brain responses to each stimulus were modeled from the onset of the target tone (i.e., final note in melodic task and 4^th^ tone in non-melodic task) based on a regressor derived from the canonical hemodynamic response function. Two analyses were done on the data. First, to remove any unwanted timbre effects, contrasts were calculated between the deviant tone and standard (in-tune or identical), separately in both melodic and non-melodic tasks (see [21,22]). Specifically, in the melodic task, we calculated an *out-of-key > in-tune* contrast and an *out-of-tune > in-tune* contrast, whereas for the non-melodic task, we calculated *6*.*25 cent deviant > identical*, *12*.*5 cent deviant > identical and 50 cent deviant > identical* contrasts. For these contrasts, we used a one-sample t-test corrected for multiple comparisons using a p-value adjusted by the false detection rate (FDR) at the group level to determine which brain regions were more active for the deviant note. These contrasts identified brain regions associated with deviance detection, separately in a melodic and a non-melodic context. While the size of the deviances are the same in both conditions, they are relative to an expected note in the melodic condition as opposed to being directly comparable to a repeated standard note in the non-melodic condition. This is an important feature of the study, as it permits the examination of how similar deviances are differentially processed in melodic and non-melodic contexts.

Next, we compared deviance detection between the melodic and non-melodic tasks. Note that the out-of-tune change in the melodic task was of the same size as the 50 cent deviant in the non-melodic task. Accordingly, we compared the 50 cent change contrast between the melodic and the non-melodic task. More specifically, for each participant we generated individual t-maps for these two contrasts (i.e., [*out-of-tune > in-tune*] vs. [*50 cents > identical*]) to identify regions unique to processing pitch deviants in melodic and non-melodic tasks. For all contrasts, we used a whole brain search (threshold: *p* < .001, Ke = 10) to identify significant regions. Clusters of at least 10 voxels where the probability of false-detection was below .05 for the entire cluster were considered significant. Only clusters located in cortical grey matter structures are reported.

## Results

### Behavioral results

**Fig 3:**
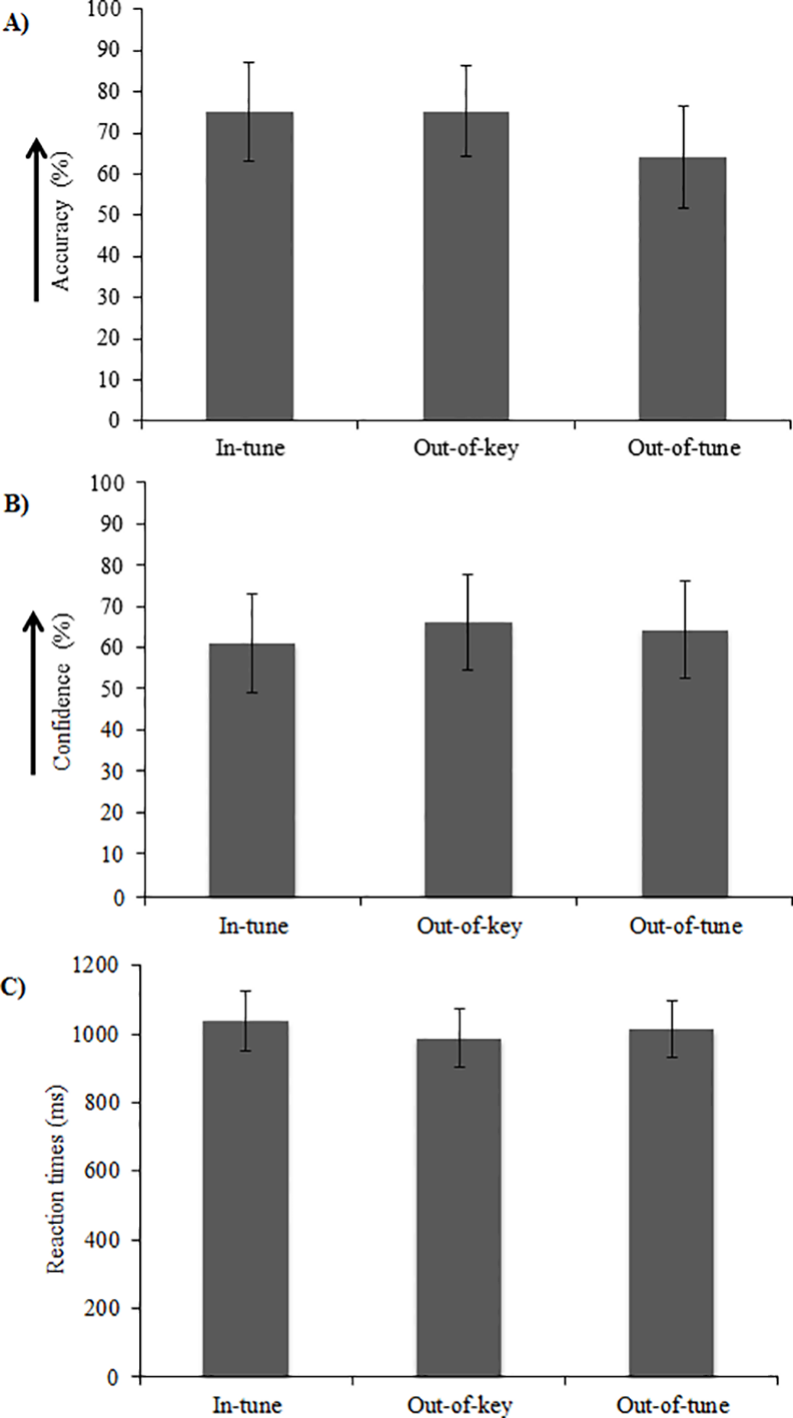
Behavioral results for the melodic task. Participants’ (A) accuracy (% correct), (B) confidence (% sure) and (C) reaction time (ms) for the melodic task. Results are presented as a function of experimental condition (i.e. in-tune, out-of-key, out-of-tune).

**Fig 4:**
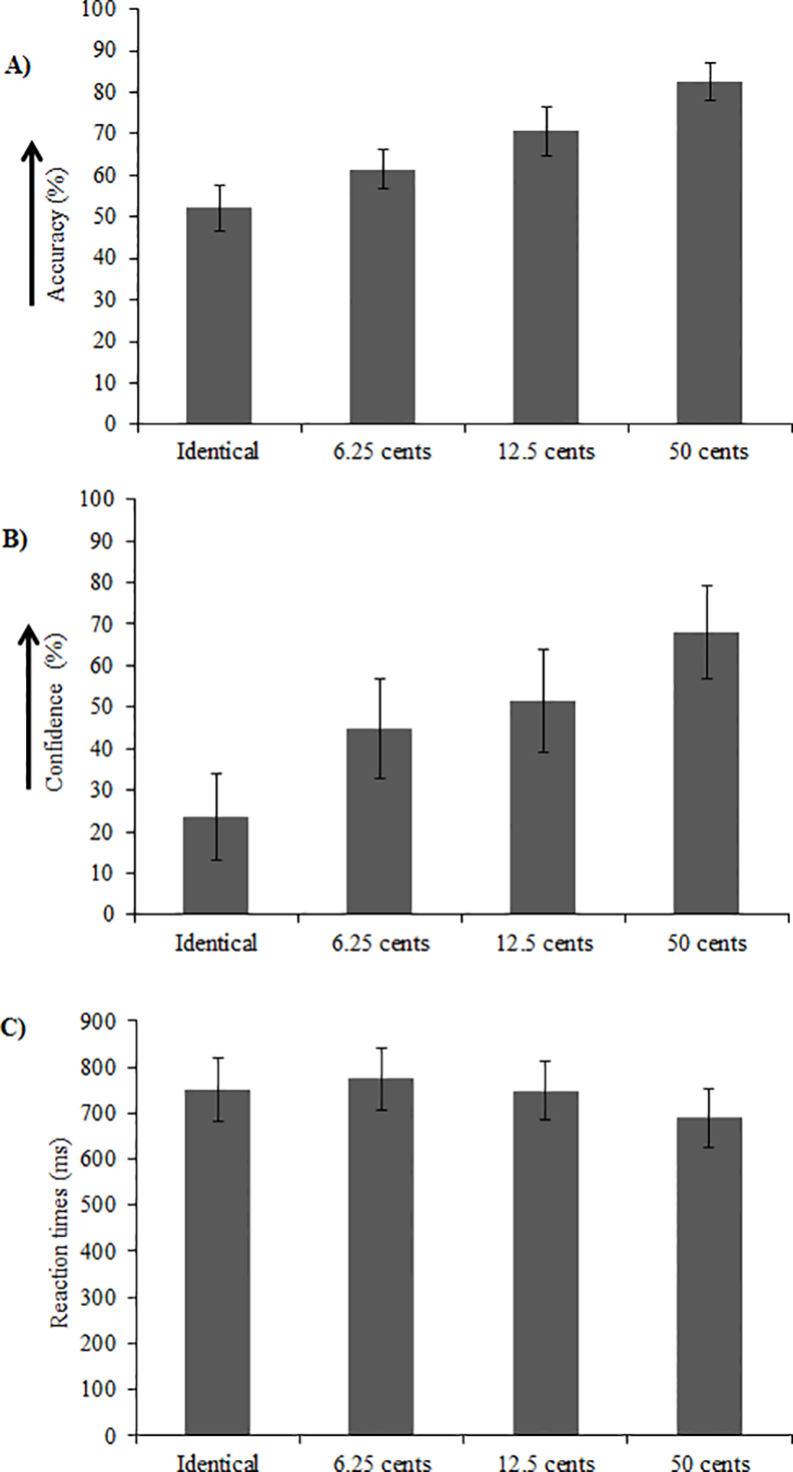
Behavioral results for the non-melodic task. Participants’ (A) accuracy (% correct), (B) confidence (% sure) and (C) reaction time (ms) for the non-melodic task. Results are presented as a function of experimental condition (i.e. Identical, 6.25, 12.50 and 50 cents). For the Identical condition in panel (A), accuracy reflects the proportion of “higher in pitch” responses, and thus accuracy of 50% is indicative of non-biased responses.

Behavioral results for the melodic task are displayed in Fig 3, whereas the results for the non-melodic task are displayed in Fig 4. Participants’ performance was assessed in terms of (A) accuracy (% correct), (B) confidence (% sure) and (C) reaction times (ms) for both the melodic and non-melodic tasks.

#### Melodic task

For the melodic task, accuracy, confidence and reaction time were each quantified using a repeated measures one-way ANOVA with stimulus type (i.e. in-tune, out-of-key, out-of-tune) as a within-subject factor (Fig 3). There was a main effect of stimulus type on accuracy, *F*(2, 22) = 3.98, *p* = .03 (Fig 3A). However, when correcting for multiple comparisons using Tukey’s HSD test, the accuracy difference were not significant. Neither confidence nor reaction time were impacted by stimulus type, *F*(2, 22) = 1.10, *p* = .35 and *F*(2, 22) = 2.14, *p* = .14, respectively (Fig 3B and 3C).

#### Non-melodic task

For the non-melodic task, accuracy, confidence and reaction time were each quantified using a repeated measures one-way ANOVA with stimulus type (6.25, 12.50 and 50 cents) as a within-subject factor (Fig 4). Data for the *identical* stimulus type was not included in this analysis because the forced choice task demanded a response of ‘higher in pitch’ or ‘lower in pitch’, thus yielding accuracy of ~0 for this condition (as there was no correct answer). In order to confirm that participants did not show a response bias in the identical condition, “higher” responses were coded as 1 and “lower” responses were coded as 0, and then a one-sample t-test against a value of 0.5 (i.e., no bias towards either response) was calculated. This test indicated that responses were not biased, *t*(12) = 1.23, *p* = .24.

In a first step, the ANOVAs for accuracy, confidence, and reaction time were run with pitch change direction (up and down) as an additional within-subjects factor. The main effect of pitch change direction was found to be not significant in all cases, nor did pitch change direction interact significantly with stimulus type (all *F* values < 6.11, all *p* values > .05). Therefore, we collapsed our data across the two pitch change direction categories for the subsequent analyses.

Accuracy increased as the pitch deviation increased, *F*(3, 36) = 22.70, *p* < 0.001 (Fig 4A). However, when correcting for multiple comparisons using Tukey’s HSD test, the accuracy differences between the different deviation conditions were not significant. Furthermore, one-sample t-tests revealed that accuracy was above chance in all deviant conditions (all *p* values < .001).

Confidence increased as the size of the pitch deviant increased, *F*(3, 36) = 18.51, *p* < .001 (Fig 4B). However, when correcting for multiple comparisons using Tukey’s HSD test, there were no significant differences in confidence between conditions.

Reaction time was shortest when pitch deviation was largest, *F*(3, 36) = 4.17, *p* = .01 (Fig 4C). Pair-wise comparisons as well as a Tukey’s HSD test confirmed that reaction times were shorter for the 50 cent deviant compared to the 6.25 deviant, *t*(12) = -3.00, *p* = .01, but no significant differences were found between the 50 cent and 12.5 deviants and between the 12.5 and 6.25 deviants (*p* = .07 and .09 respectively).

### Imaging results

**Fig 5:**
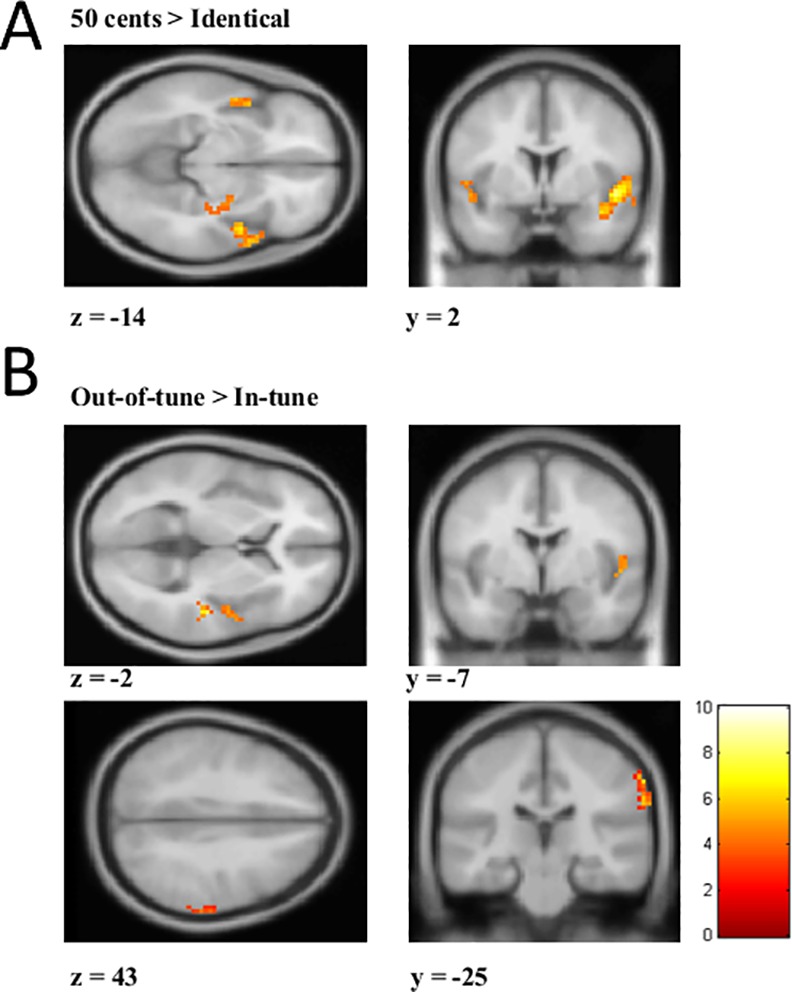
Activation maps to deviants in non-melodic and melodic contexts. (A) Activation map for the *50 cents > Identical* contrast from the non-melodic task. A mask was applied to highlight activation in the superior temporal gyrus (STG) for visualization purposes. (B) Activation map for the *Out-of-tune > In-tune* from the melodic task. In the top image, a mask was applied to highlight activation in the STG, and in the bottom image, a mask was applied to highlight activity in supramarginal gyrus/inferior parietal lobule (IPL). Scale represents t-values.

**Fig 6:**
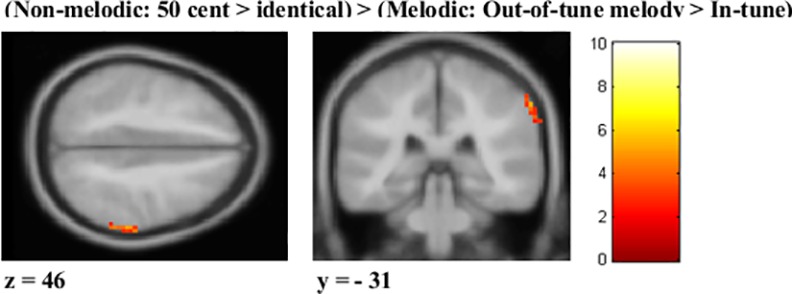
Brain regions responding more strongly to the melodic task compared to the non-melodic task. Activation map for the (**Non-melodic**: *50 cents > identical*) > (**Melodic**: *Out-of-tune > In-tune*). A mask was applied to highlight activation in the supramarginal gyrus/inferior parietal lobule (IPL) for visualization purposes. Scale represents t-value.

Imaging results are presented in Tables 1–4 and Figs 5 and 6. Voxel sizes were 3 X 3 X 3.2 (28.8 mm^3^). For the melodic task, out-of-tune melodies elicited greater activity in the right STG and IPL compared to in-tune melodies (Fig 5B, Table 1). For the out-of-key melodies, no clusters met our statistical criteria. For the non-melodic tasks, both 50 cent and 12.5 cent deviants evoked greater activity in the STG, cingulate, and other areas (Fig 5A, Tables 2 and 3). For the 6.25 cent deviant, no clusters met our statistical criteria. Regions where neural activity was greater for melodic deviance detection compared to non-melodic deviance detection are presented in Table 4 and Fig 6. The only significant region was the inferior parietal lobule/supramarginal gyrus. There were no regions where neural activity was significantly greater for the non-melodic task compared to the melodic task.

**Table 1:** Melodic task: *Out-of-tune > In-tune*.

Region	Cluster size (k)^a^	X,Y,Z	T(peak)	P (FDRcorr)^b^
Right Supramarginal, IPL	22	66, -25, 43	6.2	.062
Right STG	31	48, -7, -2	5.63	.034

^a^ For each contrast, clusters at least 10 voxels (k) were first identified by SPM using a whole brain search where p < .001, for the cluster

^b^ Each cluster was considered to be significant if its false detection rate probability (P fdr) was below .05

**Table 2:** Non-melodic task: *50 cents > Identical*.

Region	Cluster size (k)^a^	X,Y,Z	T(peak)	P (FDRcorr)^b^
Right STG	417	39, 5, -20	8.42	< .001
Left STG	55	-48, -1, -8	6.06	.003
Left Cingulate gyrus	37	-3, 2, 46	5.87	.017
Left Hippocampus	33	-33, -13, -14	6.67	.022
Left Fusiform Gyrus	27	-33, -46, -14	6.31	.039

^a^ For each contrast, clusters at least 10 voxels (k) were first identified by SPM using a whole brain search where p < .001, for the cluster

^b^ Each cluster was considered to be significant if its false detection rate probability (P fdr) was below .05

**Table 3:** Non-melodic task: *12*.*5 cents > Identical*.

Region	Cluster size (k)^a^	X,Y,Z	T(peak)	P (FDRcorr)^b^
Right STG	67	69, -31, 7	7.86	.002
Left STG	28	-48, -4, -8	5.88	.042
Left Superior Parietal Lobule	37	-27, -40, 52	7.4	.019
Supplementary motor area (L&R)	89	-12, 5, 49	7.36	.001
Left Middle Frontal Gyrus	71	-27, -13, 58	6.8	.002
Left Insula	25	-30, 11, 10	6.46	.053
Right Cingulated Gyrus	38	15, -31, 28	6.4	.019
Right Pre-central gyrus white matter	39	21, -13, 55	6.25	.019
Right Postcentral gyrus	47	48, -22, 37	5.54	.011

^a^ For each contrast, clusters at least 10 voxels (k) were first identified by SPM using a whole brain search where p < .001, for the cluster

^b^ Each cluster was considered to be significant if its false detection rate probability (P fdr) was below .05

**Table 4:** Melodic pitch discrimination (Melodic [*Out-of-tune > In-tune*] > Non-melodic [*50 cents > Identical*]).

Region	Cluster size (k)^a^	X,Y,Z	T(peak)	P (FDRcorr)^b^
Right Supramarginal/ IPL	17	63, -31, 46	5.81	.016

^a^ For each contrast, clusters at least 10 voxels (k) were first identified by SPM using a whole brain search where p < .001, for the cluster

^b^ Each cluster was considered to be significant if its false detection rate probability (P fdr) was below .05

In order to examine whether the observed right IPL activation was related to task difficulty, additional contrasts were performed in the non-melodic task. Using the same imaging parameters, the (difficult) 12.5 cents > (easy) 50 cents contrast did not yield any clusters meeting the statistical criteria. Thus, it is unlikely that the observed right IPL activation reflects a difficulty effect.

## Discussion

The present study compared pitch change detection in two distinct contexts: (1) a melodic context and (2) a non-melodic context. The purpose was to determine which brain areas are activated uniquely or preferentially by deviants in the musical context, which theoretically requires the processing of musical structure in addition to pitch change detection. Consistent with our hypotheses and previous research, we found significant activation in the right superior temporal gyrus (STG) during pitch violations in both melodic and non-melodic contexts, (e.g. [3,5]). Only the right IPL was significantly more active in the melodic task compared to the non-melodic task, and this activation was limited to out-of-tune deviants. This comparison required the use of naturalistic materials to reflect the information available to listeners during music listening. Accordingly, our findings suggest a hemispheric asymmetry for pitch processing in a musical context, where right temporo-parietal structures show preferential processing for the evaluation of pitch information in terms of tonal structure.

### Brain areas underlying general pitch processing and discrimination

Past research on brain responses to pitch deviants suggest a key role for the auditory areas of the STG and frontal cortices [23,24]. In both the melodic and the non-melodic contexts, the presence of deviant notes increased activation within the STG. Several studies have shown similar temporal activations during pitch discrimination tasks in a melodic and non-melodic setting [20,25,26]. Using fMRI in humans, Norman-Haignere et al. [27] have shown that pitch-sensitive brain regions exhibited an overall preference for resolved harmonics compared to unresolved harmonics and noise, and that pitch responses were biased towards anterior regions of the superior temporal plane of the auditory cortex. Barker et al. [28] also showed greater pitch-related activation in a pre-defined pitch-responsive region, encompassing parts of the central and lateral Heschl’s gyrus and planum temporale, than in the medial Heschl’s gyrus for pitch and slow spectrotemporal variations as compared to baseline Gaussian noise, further implicating these regions in pitch processing. The STG has long been known to be an important region for pitch processing [29,30]. The lack of activation observed in frontal regions during pitch discrimination in the present study was surprising, and may have been due to the high rate of stimulus presentation, as well as the fact that deviant and non-deviant stimuli were presented randomly, rather than within blocks. The STG likely responds quickly to a changing environment as it processes successive auditory inputs. In contrast, the frontal regions are involved in monitoring and interpreting the deviant in order to initiate a behavioural response, and thus might operate differently in time (e.g. [5,31]). This could result in overlapping frontal activity between trials and a lack of difference between deviant and non-deviant trials.

### Music specific processing

The right IPL was the only region uniquely activated by the melodic task, and this activation was elicited by one specific kind of deviants in the melodic task, namely the out-of-tune deviants. The IPL is active when comparing melodies that are identical, but transposed [32], and when comparing melodies that differ in terms of their melodic structure or rhythm [33]. Consequently, the IPL has been frequently associated with tonal working memory. Tonal working memory allows for the storage and manipulation of information and facilitates the comparison of melodies [34,35]. For instance, Koelsch and colleagues [34] presented participants with strings of sung syllables, and showed that rehearsal of verbal as well as tonal information activated a network of brain areas including the IPL. Interestingly, there is considerable overlap between verbal and tonal working memory (e.g. [20,34–36]). In the current study, no verbal task was performed, thus, the IPL activation unique to the melodic task likely underlies working memory processes that are related to comparing incoming acoustic information to a representation of the musical context.

Support for this hypothesis comes from Foster and Zatorre [32], who showed that the right intraparietal sulcus (IPS) was activated by a transposed melody discrimination task, but not a simple melodic comparison. The transposition task involved a four-semitone transposition between the target and comparison patterns, and thus required listeners to use interval information rather than the absolute value of the individual pitches in the melody to make the comparison. Furthermore, the activity within the right IPS predicted task performance for both musicians and nonmusicians in the transposed melody condition. The authors concluded that the IPL played a role in the relative pitch computation required by the task, and more importantly likely plays an important role in transforming high-level melodic auditory information. Further support for this proposal comes from other studies that have found activation near the IPL for other mental musical transformations [32,37]. For example, the IPS was activated when asking participants to mentally reverse a melody [37]. Additionally, a conjunction analysis revealed that the right IPS was active for both the musical mental reversal task and the transposed melody discrimination task described above, highlighting the IPS’ role in musical transformations.

In the current study, participants had to maintain the key of each melodic sequence in working memory in order to be able to detect deviations. Our data indicate that integrating deviant events into the ongoing tonal context is supported by activity in the IPL. Unlike previous studies, which have used musical chords, the current task used monophonic melodies, which require time to establish a tonality. Interestingly, when comparing brain activations between tonic chords (most structurally important) and subdominant chords (less structurally important) placed at the end of a chord sequence, Tillmann et al. [38] found increased activation in the supramarginal gyrus. The peak of this activity was inferior to the IPL/supramarginal peak observed in the melodic task in the current study. In the case of Tillmann et al.’s study [38], each chord could establish a tonal structure, whereas in the current study a few tones are needed in order to establish a musical key. Therefore, the overall pattern of results suggests that more superior portions of the IPL are involved in establishing a tonal center from melodies that unfold over time. However, here IPL activation was observed for out-of-tune tones and not for the out-of-key notes. Melodically, out-of-key notes are sometimes used by composers to add tension and complexity to their work. In contrast, out-of-tune notes are rarely used, and would be difficult to perform on many instruments, like a piano. In other words, an out-of-key note violates the diatonic scales but may be part of the melody because it is a legal note (i.e., part of the chromatic scale), whereas the out-of-tune note is unlikely to be part of any conventional melody in the Western tonal system. Thus, the out-of-tune note could possibly have been perceived as more salient. Accordingly, the observed activity in the right IPL is likely associated with a working memory process that is tracking tones for tonal incongruency [39].

One limitation of the current study was the difference in the proportion of non-deviant trials in the melodic and non-melodic tasks. In the melodic task half of the presented melodies were non-deviants, while in the non-melodic task only 25% were non-deviants. Additionally, the melodic task covered a wider frequency range than the non-melodic task, which could have led to stronger adaptation in the non-melodic task. Finally, the nature of the behavioral tasks also differed slightly between the conditions (i.e. congruence judgment vs. up-down discrimination), which might have had an unavoidable effect on task difficulty. Because the melodic task involved more cognitive or higher-level processing, the difficulty might always be greater for this type of task compared to a non-melodic task. Therefore, even if deviant magnitudes were to be adjusted to achieve equivalent accuracy or reaction times, low-level differences would still remain.

To overcome these limitations, we compared brain activation between the melodic and non-melodic tasks using within-task contrasts that were calculated first. By comparing the within-task standard note (i.e., in-tune/identical) with the deviant (out-of-key/out-of-tune; different), we controlled for any stimulus specific effects in each task and isolated neural processes related only to processing the deviant note. Furthermore, comparing the “difficult” to the “easy” conditions in the non-melodic task did not reveal significant IPL activation, and there was no significant difference in accuracy between the melodic and non-melodic tasks. Therefore, the IPL activation seen for the out-of-tune deviants in the melodic context is unlikely to reflect a difficulty effect. Nonetheless, future studies should be careful in adequately matching task difficulty in order to avoid this potential confound.

Additionally, the possibility that stimulus-specific adaptation might have played a role in the recorded brain activity patterns cannot be excluded. The idea that neural responses to deviant sounds can be explained by neural adaptation is not new. For instance, Jääskeläïnen et al. [40] proposed that the mismatch negativity (the ERP response to auditory deviants) could be explained by neural adaptation (indexed by subtracting the N1 to standard sounds from those to deviant sounds). However, Näätänen et al. [41] have described multiple reasons that the MMN must be generated at least in part by a neural population that is distinct from those neurons that generate the N1 response. Namely, the MMN latency and duration do not match that of the N1, the MMN can be recorded in the absence of the N1 and feature-specific adaptation, the scalp distribution of the MMN does not match that of the N1, the MMN and N1 are differentially sensitive to a variety of experimental manipulations, and the behavioural sensitivity to changes in frequency exceeds what can be predicted by the relatively wide receptive fields of N1 generating neurons.

Related to this last point, the stimuli used in the present study activated large portions of the auditory frequency map, and the deviant notes largely overlapped with that region. This overlap in frequency is such that the deviant notes never fell in a critical band that had not already responded to other stimuli, making it unlikely that they activated non-habituated afferences [41,42]. Nonetheless, stimulus-specific adaptation may still be a neural mechanism involved in the pitch-discrimination tasks.

## Conclusion

The right IPL was found here to preferentially process out-of-tune pitch violations in melodic contexts relative to non-melodic contexts. Increased activation in the IPL likely reflects the maintenance and manipulation of incoming pitch information in terms of tonal structure, this suggests that right temporo-parietal structures are implicated in the processing of tonal violations in music.
